# Meal Frequency and Body Composition in Obese Adults Presenting for a Weight-Loss Program: A Cross-Sectional Study

**DOI:** 10.7759/cureus.105105

**Published:** 2026-03-12

**Authors:** Andrew El Alam, Mohamad Fleifel, Bertha Maria Nassani

**Affiliations:** 1 Endocrinology, Diabetes, and Metabolism, Lebanese American University Medical Center, Beirut, LBN; 2 Endocrinology, American University of Beirut Medical Center, Beirut, LBN; 3 Internal Medicine and Clinical Immunology, Lebanese American University Medical Center, Beirut, LBN

**Keywords:** dietary fiber, management of obesity, nutrition and metabolism, obesity, obesity and nutrition, obesity counseling, weight loss and obesity

## Abstract

Background: Meal frequency takes a large focus when people enter a weight-loss program.

Objective: The primary aim of this study was to compare fat percentage and muscle percentage across three self-reported meal-frequency patterns, including small frequent meals (SFM), three regular meals (3RM), and three large meals (3LM), in adults with obesity enrolled in a weight-loss program at Louis Pasteur Hospital between November 2022 and June 2024. We hypothesized that meal frequency would not be associated with differences in body-composition outcomes after adjustment for age and sex.

Methods: This is a cross-sectional study of 320 patients with obesity presenting to a program for weight loss. Body mass composition was obtained, and fat and muscle percentages were calculated. Differences were compared based on meal frequency using analysis of covariance (ANCOVA) while adjusting for age and sex.

Results: A total of 320 patients were included, with meal frequency distributed as SFM (n = 153), 3RM (n = 95), and 3LM (n = 70). The mean fat percentage was 47.1, 46.3, and 46.1 (p = 0.586) for SFM, 3RM, and 3LM, respectively. The mean muscle percentage was calculated as 49.4, 51, and 51.1 (p = 0.142) for SFM, 3RM, and 3LM, respectively.

Conclusion: Eating pattern is not associated with altered body mass composition in patients presenting for weight loss.

## Introduction

Meal frequency is usually given much focus when counseling patients for weight management, although there is conflicting evidence regarding the superior body-composition outcomes of “small, frequent meals.” Higher feeding frequency is associated with lower fat mass and greater fat-free mass, according to a meta-analysis by Schoenfeld et al. (2015); however, only one study identified this effect according to sensitivity analysis, which reduces the confidence in a real benefit [1]. Increasing meal frequency under controlled energy intake typically does not result in greater weight or body composition improvements in randomized trials involving adults with obesity [2]. Furthermore, research conducted in carefully regulated environments has not demonstrated that eating more frequently improves 24-hour fat oxidation [3]. According to professional statements, increasing meal frequency does not positively alter body composition in sedentary populations [4]. We examined real-world data from obese adults who presented to the Louis Pasteur Hospital weight-loss program, comparing fat percentage and muscle percentage across three self-reported eating patterns, in light of these uncertainties and the clinical significance of maintaining muscle while reducing fat during obesity care.

## Materials and methods

This is a cross-sectional, observational, retrospective study consisting of 320 adults with obesity who presented for enrollment in a structured weight-loss program at Louis Pasteur Hospital, between November 2022 and June 2024.

Inclusion criteria were patients with BMI >30 kg/m^2^ and age above 18 years, who had complete anthropometry and body-composition data, and who had clearly reported their eating patterns. Patients who reported alternating or non-clear eating patterns were excluded from this study, in addition to patients with missing body composition data.

Obesity was defined as BMI > 30 kg/m^2^. All patients participating had baseline anthropometry and body composition measured using a bioelectrical impedance analysis (BIA) device (InBody S10/770, InBody Co., Ltd., Seoul, Korea). Measurements were done based on routine clinical assessments after overnight fasting.

Three eating patterns were identified: small frequent meals (SFM), three large meals (3LM), and three regular meals (3RM). Eating patterns were self-reported by the patients and classified by a dietician, based on the category that best represented the intake of the patients.

From recorded fat mass (kg), muscle mass (kg), and weight (kg), we computed fat mass percentage and muscle mass percentage as follows:

Fat mass percentage (fat%) = fat mass/weight × 100.

Muscle mass percentage (muscle%) = muscle mass/weight × 100.

Statistical analyses were carried out using Python version 3.11 (Python Software Foundation, Wilmington, Delaware). Group differences in fat percentage and muscle percentage were examined using one-way analysis of variance (ANOVA) with η² (eta squared) as the effect size. Assumptions were verified, and Kruskal-Wallis tests were used for confirmation. Because age differed between groups, analysis of covariance (ANCOVA), adjusting for age and sex, was also performed. A two-sided α of 0.05 was considered significant. No other potential confounders were considered. Overall group comparisons for either fat percentage or muscle percentage were not statistically significant, not necessitating post hoc tests.

## Results

The total sample size was 320. The mean age was 43.8 ± 12.6 years. Sex distribution was 197 females and 123 males. Group sizes were as follows: SFM = 153, 3RM = 95, and 3LM = 72. Age varied according to eating habits, with patients in the 3RM group being older on average (F = 5.54, p = 0.004) (Table 1).

**Table 1:** Baseline characteristics of the study population by meal frequency group. SFM: small frequent meals; 3LM: three large meals; 3RM: three regular meals.

Characteristic	Value
Total sample size	320
Mean age ± SD (years)	43.8 ± 12.6
Females, n (%)	197 (61.6%)
Males, n (%)	123 (38.4%)
SFM, n	153
3RM, n	95
3LM, n	72
Age difference by group (F, p)	5.54 (0.004)

Body-composition percentages by eating habit

Muscle Percentage

**Figure 1:**
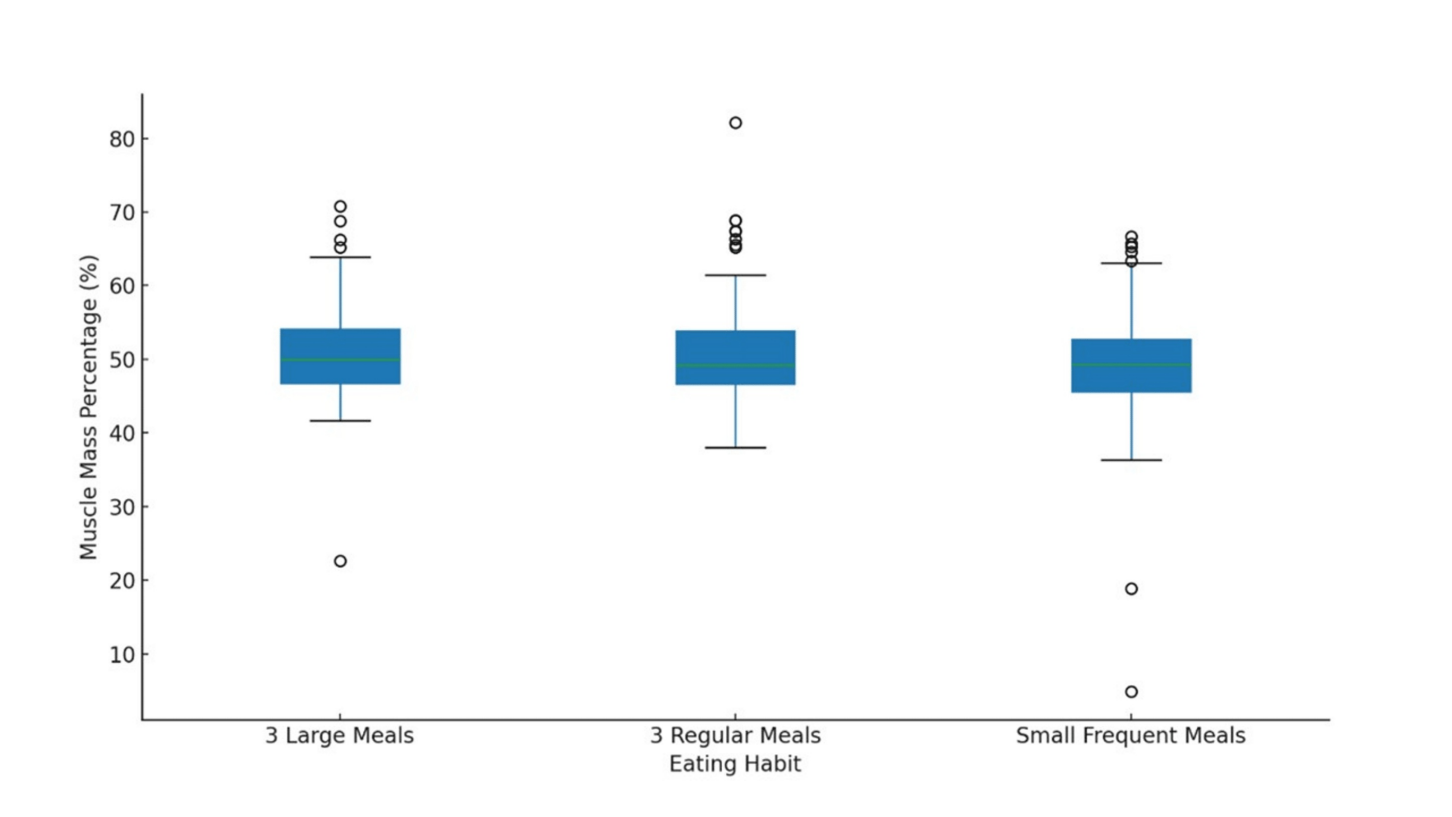
Distribution of muscle mass percentage by eating habits.

Muscle percentage did not differ significantly between meal-frequency groups (SFM: 49.4 ± 7.5%; 3RM: 51.0 ± 7.2%; 3LM: 51.1 ± 7.3%). One-way ANOVA (F = 2.321, p = 0.100, η² = 0.012) indicated a very small effect size. After adjustment for age and sex, ANCOVA showed no significant effect of meal pattern (F = 2.321, p = 0.100). Assumptions of normality and homogeneity of variance were satisfied (Figure 1).

Fat Percentage

**Figure 2:**
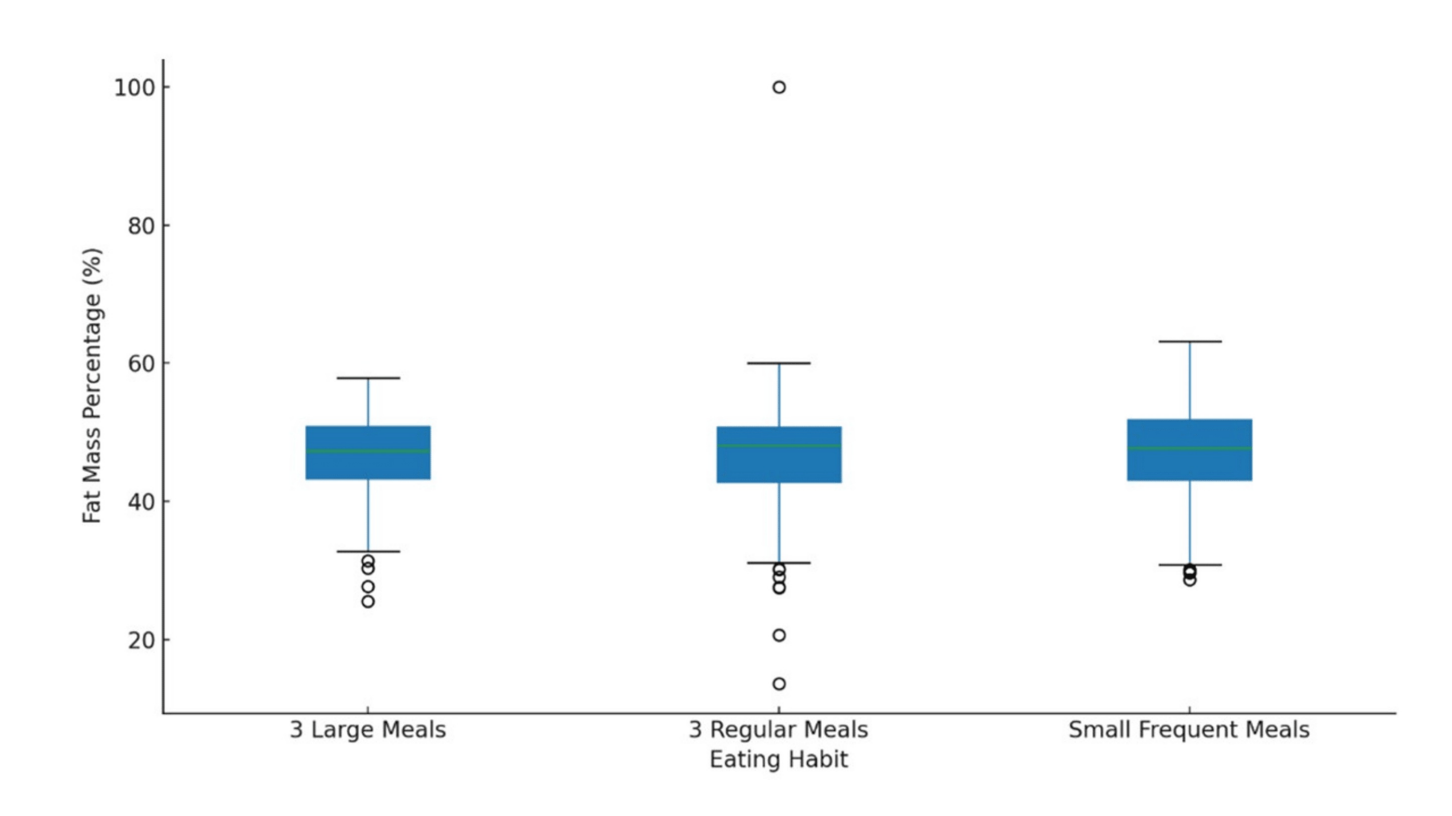
Distribution of fat mass percentage by eating habits.

There was no significant difference between groups with regard to fat percentage (SFM: 47.1 ± 7.0%; 3RM: 46.3 ± 9.8%; 3LM: 46.1 ± 7.0%). One-way ANOVA (F = 0.504, p = 0.604, η² = 0.003) indicated a negligible effect size. After adjusting for age and sex, ANCOVA showed no significant effect of meal pattern (F = 0.504, p = 0.604). Assumptions were met concerning the normality and homogeneity of variance (Figure 2).

**Figure 3:**
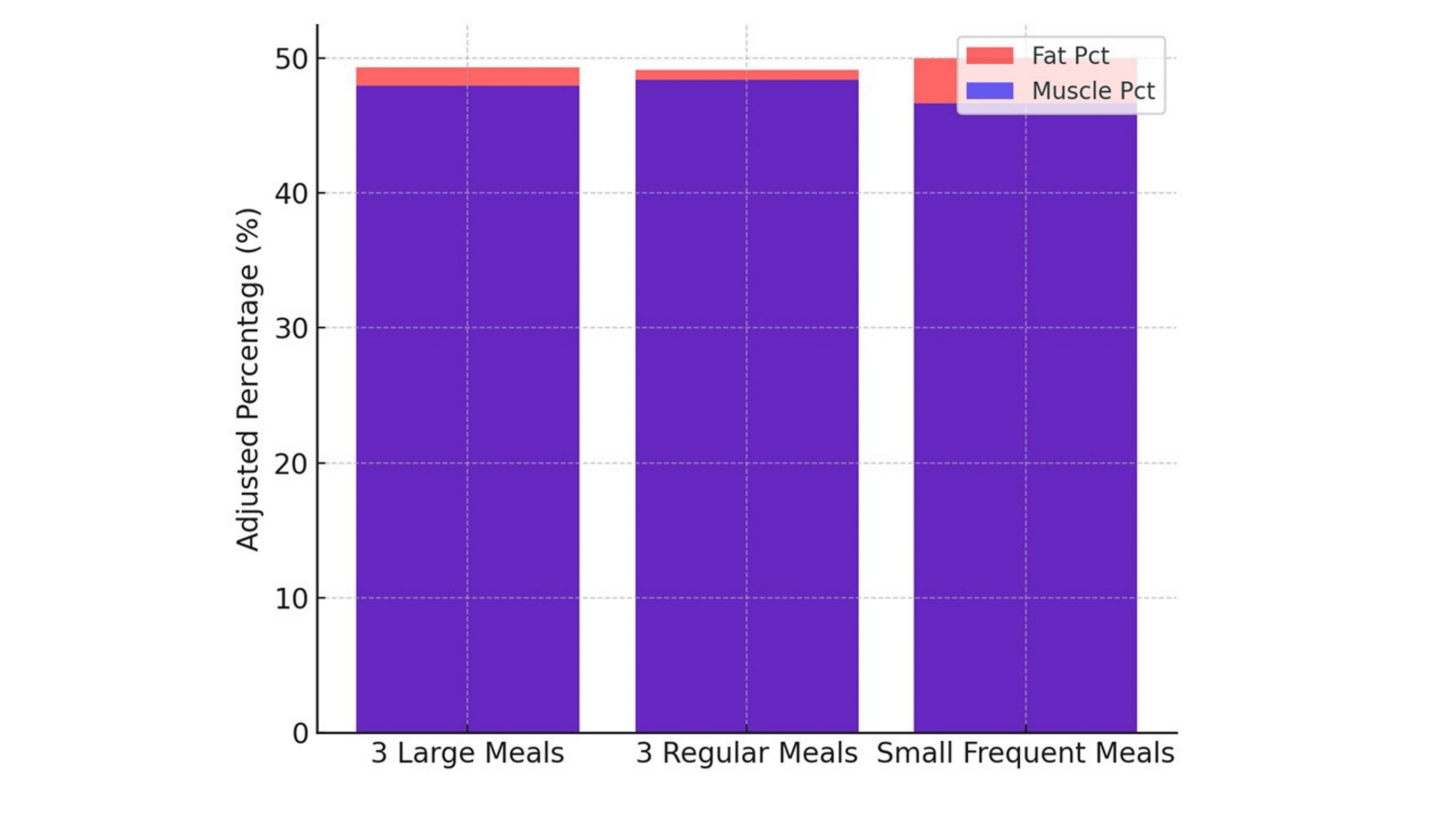
Age- and sex-adjusted mean muscle percentage and fat percentage by eating habits.

Across all tests, no statistically significant differences emerged for fat percentage or muscle percentage between SFM, 3LM, and 3RM (Figure 3).

## Discussion

In this cross-sectional study of obese patients seeking treatment, the results outlined that there is no significant association between eating habit category and fat percentage or muscle percentage. These findings align with randomized controlled trials (RCTs) showing that increasing meal frequency does not lead to better weight or body composition changes when the energy intake is matched. For example, Cameron et al. (2010) randomized participants to higher vs. lower meal frequency under an energy-restricted diet. There was no advantage of higher frequency for weight loss or body composition [2]. Another one-year RCT in obese patients that compared three meals/day and three meals plus three snacks per day reported no difference in weight loss between the two [3]. A controlled whole-room calorimeter study, with isoenergetic balanced diets, showed no improvement when there was an increase from three to six meals/day [4]. A meta-analysis (2015) showed benefits caused by higher meal frequency on body composition, but the apparent advantage was based on a single study according to sensitivity analysis [1]. Position statements of the International Society of Sports Nutrition conclude that increasing meal frequency alone is unlikely to improve or change body composition [5]. Our findings, which show null results (η² ≈ 0.003-0.012), support this larger body of literature. For patients with obesity, what and how much a person eats, with the distribution of proteins across meals, may be more helpful for muscle building than simply increasing meal count. A study by Mamerow et al. (2014) showed that distributing protein evenly across meals better enhanced muscle protein synthesis than concentrating protein intake with the evening meal [6]. This supports our findings, which showed no increase in muscle bulk. Our findings are further supported by a recent systematic review with meta-analysis of RCTs by Blazey et al. (2023), which found no clear difference between a high or a low eating frequency on changes in fat mass or other body-composition outcomes in adults [7]. Recent evidence from a systematic review conducted for the 2024 Dietary Guidelines Advisory Committee found limited and inconsistent evidence linking the number of daily eating occasions with body composition or obesity outcomes in adults [8]. Our study not only shows comparable results with previous studies and trials, but also describes a real issue of conflict that occurs in every weight loss clinic. A large focus on the frequency of meals is given in practice for the attempt at weight loss and muscle bulking, while real evidence-based methods are not tackled properly. This article emphasizes the importance of education on the timing of protein intake, caloric count, and structured physical activity in fighting obesity and changing body composition.

Strengths and limitations

Our study has its limitations, as it was conducted in a single center with self-reported meal patterns that could lead to misclassifications. There is an absence of dietary caloric data and physical activity. Another limitation of this study is its cross-sectional design, which precludes inference of a cause-and-effect relationship between meal frequency and body-composition outcomes. Its strengths include a clinically relevant treatment-seeking obese cohort and standardized body-composition measurements obtained under optimal conditions.

## Conclusions

In this cross-sectional study of an obese adult population presenting to a weight-loss program, the meal frequency patterns showed no association with the differences in fat percentage and muscle percentage. The findings suggest that, in this population seeking treatment, meal frequency alone may not be a strong determinant of body composition differences.

Given the cross-sectional design, relying on self-reported eating patterns, in addition to the absence of data on calorie intake and physical activity, we cannot establish a causal relationship. Focus should be on future prospective and randomized control trials to further evaluate the role of meal frequency in body composition among patients with obesity. In real practice, efforts should be placed on overall dietary quality, caloric balance, adequate protein intake, and physical activity rather than meal frequency alone.
